# Time Effectiveness of Ultraviolet C Light (UVC) Emitted by Light Emitting Diodes (LEDs) in Reducing Stethoscope Contamination

**DOI:** 10.3390/ijerph13100940

**Published:** 2016-09-23

**Authors:** Gabriele Messina, Mattia Fattorini, Nicola Nante, Daniele Rosadini, Andrea Serafini, Marco Tani, Gabriele Cevenini

**Affiliations:** 1 Laboratory of Environmental Hygiene, Department of Molecular and Developmental Medicine, University of Siena, Via Aldo Moro 2, 53100 Siena, Italy; nicola.nante@unisi.it; 2 Post Graduate School of Public Health, University of Siena, Via Aldo Moro 2, 53100 Siena, Italy; fattorini7@student.unisi.it (M.F.); daniele.rosadini@student.unisi.it (D.R.); serandre85@gmail.com (A.S.); 3 FeT Elettronica S.n.c Via A. Volta 28, 53036 Poggibonsi (Siena), Italy; mtani@fet.it; 4 Department of Medical Biotechnologies, University of Siena, Via Aldo Moro 2, 53100 Siena, Italy; gabriele.cevenini@unisi.it

**Keywords:** disinfection, ultraviolet, LED, stethoscope, health-care associated infections

## Abstract

Today it is well demonstrated that stethoscopes can be as contaminated as hands, which are a recognized source of Health-Care Associated Infections (HCAIs). Ultraviolet C (UVC) light has proven disinfection capacity and the innovative UVC technology of Light Emitting Diode (LED) shows several potential benefits. To verify whether the use of UVC LEDs is effective and reliable in stethoscope membrane disinfection after prolonged use, a pre-post intervention study was conducted. A total of 1668 five-minute cycles were performed on two UVC LEDs to simulate their use; thereafter, their disinfection capacity was tested on stethoscope membranes used on a previously auscultated volunteer. Then, a further 1249 cycles were run and finally the LEDs were tested to assess performance in reducing experimental contamination by *Staphylococcus* *aureus*, *Pseudomonas* *aeruginosa* and *Escherichia coli* on the stethoscope membrane. Baseline volunteer contamination identified 104 Colony Forming Units (CFUs) while treated Petri dishes had 12 and 15 CFUs (*p* < 0.001). Statistically significant differences (*p* < 0.001) were also found relating to the reduction of specific bacteria: in particular, after treatment no CFU were observed for *S. aureus* and *E. coli*. UVC LEDs demonstrated the capacity to maintain high levels of disinfection after more than 240 h of use and they were effective against common microorganisms that are causative agents of HCAIs.

## 1. Introduction

According to the World Health Organization (WHO), Health-Care Associated Infections (HCAIs) *«[…]are infections that patients acquire while receiving treatment for medical or surgical conditions and are the most frequent adverse event during care delivery»* [1]. Zimlichman et al. [2] evaluated that the five most common types of HCAIs (surgical site infections, central line-associated infections, catheter-associated infections, ventilator-associated pneumonia and *Clostridium difficile* infections) have an annual economic impact of $9.8 billion in the U.S. It is also estimated that up to 32% of HCAIs can be prevented [3]. It is widely reported that environmental surfaces and medical devices make a substantial contribution to the transmission and diffusion of HCAIs in healthcare settings [4,5,6,7]. According to Weber et al. [5], 20%–40% of HCAIs could be caused by pathogens hosted on the hands of Health-Care Workers (HCWs), which may become contaminated after contact with inanimate surfaces. Moreover, it has been reported that certain pathogens (e.g., Methicillin-resistant *Staphylococcus aureus*, Vancomycin-resistant *Enterococcus* and *C. difficile*) could even survive for a month on environmental surfaces and medical devices [5,8]. Although it is difficult to highlight a direct association between stethoscope contamination and the onset of HCAIs [3], microbial contamination of stethoscopes has already been demonstrated, and this may contribute in spreading of pathogens involved in HCAIs. In addition, it has also been demonstrated that the level of contamination is comparable to that of the physician’s dominant hand, one of the leading causes of HCAIs [3,9]. Studies have shown the presence of the same bacterial strain both in patients and on stethoscopes previously used for their auscultation [10,11]. Hence, stethoscope disinfection gains remarkable importance, although surveys show that up to 90% of physicians do not disinfect their stethoscope after performing examinations [9]. This poor compliance among HCWs could be explained by various factors, such as lack of prompt cleaning agents and high workload leading to forgetfulness but, if informed, HCWs do realize the importance of cleaning the stethoscope [12]. The use of Ultraviolet C light (UVC) emitted by Light Emitting Diodes (LEDs) has been already proposed and tested as a method for obtaining disinfection of the stethoscope membrane [13], but considering the early adoption of this technology and the shorter operative life of UVC LEDs than standard ones, to our knowledge, there is a lack of data concerning the effectiveness of LEDs after prolonged use. The aim of the study is to verify whether, after extended use, UVC LEDs remain effective in significantly reducing stethoscope membrane contamination both on the skin and in a laboratory setting.

## 2. Materials and Methods

### 2.1. Setting

**Figure 1:**
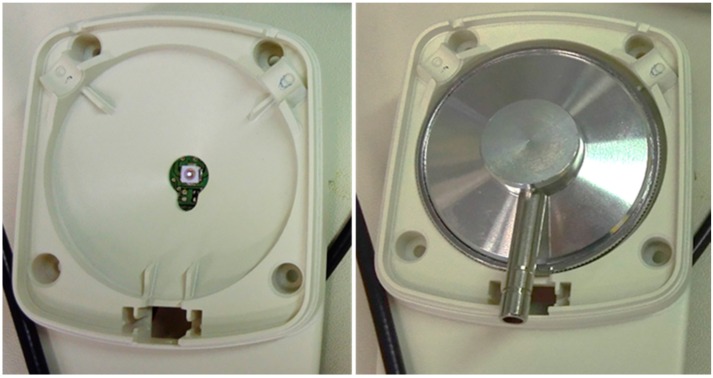
Experimental prototype developed for the execution of the experiments. On the right, the correct positioning of the stethoscope head during Ultraviolet C light (UVC) disinfection is shown.

An experimental design with pre-post treatment was conducted between August 2015 and March 2016 in the Laboratories of the Department of Molecular and Developmental Medicine, University of Siena. Using a 3D printer (Sketchup, Boulder, CO, USA) we created an experimental prototype for properly placing LEDs and the stethoscope head (Figure 1).

Two UVC LEDs (LED 16 and 18) produced by SeoulViosys (Ansan-si, Gyeonggi-do, Korea), (model CUD8DF1A, 275 nm wavelength-peak, average power 2.4 mW) were used to irradiate a stethoscope head inserted in the prototype, at a distance ranging from 11 to 23 mm, in the center and edges of the membrane respectively. Implementing a battery power supply and a microcontroller, we were able to run repeatable cycles using 5 min of irradiation followed by 1 min of inactivity. Each cycle of functioning corresponded to one disinfection procedure after stethoscope auscultation of a patient. The irradiation time of 5 min was chosen in accordance with previous laboratory tests based on several factors: UV LED power, distance from the LED and irradiation target, UV susceptibility of the different bacteria [14] and factors reducing UV effectiveness (sweat, sebum). Taking into account the more resistant bacteria among those most commonly found on the skin, a rough calculation of the time sufficient for a 2 log reduction of the bacterial contamination was approximately 2–3 min. Therefore, we chose a time of 5 min in order to overcome additional obstacles such as partial overlapping of bacteria, sebum, sweat, other biological fluids, etc. The whole study was conducted in rooms with air conditioning, at a mean air temperature of 23 °C.

### 2.2. Experimental Protocol

To test the effectiveness of LEDs in reducing microbial contamination after prolonged use, we decided to organize the study into four consecutive phases.

Firstly, to simulate previous prolonged use of the LEDs, we ran them for 1668 cycles of activity, i.e., a total of 8340 min.

Secondly, to assess whether the LEDs were able to reduce microbial load on stethoscope membrane after contact with the skin, three repeated auscultations on a volunteer (with a total of ten contact points each one) were performed. In particular, the first auscultation was used as a control, while the following two were performed to test the disinfection capability of LEDs 16 and 18. Before and after each use of the stethoscope, the membrane was disinfected with alcohol to assure the same baseline setting, and dried to prevent potential inhibition of the bacterial growth by alcohol. The volunteer was a member of the Department of Molecular and Developmental Medicine of the University of Siena. He was not informed about the procedure of the study, in order to avoid modifications in personal hygiene. However, at time of auscultation, the volunteer accepted enrolment. The ten contact points of the stethoscope membrane during each auscultation were located on the thorax of the volunteer, on the front and the back: each auscultation was performed in the same part of the body, avoiding the identical points previously tested in order to prevent possible contamination due to the microbial removal caused by preceding contacts. Stethoscope membrane, after the control auscultation and the two irradiations with UVC LEDs, was applied to 60 mm Petri plates containing Plate Count Agar (PCA), and then cultured. 

In the third phase of the study, in order to simulate additional prolonged use of the LEDs we ran 1249 further cycles (6245 min) on both of them, reaching an overall 2917 cycles of functioning (14,585 min).

Thereafter, in the fourth phase, we decided to test their effectiveness in reducing the microbial load of specific microorganisms. Contamination of stethoscope membranes was performed in a laboratory, using three different bacteria: *Staphylococcus aureus* (ATCC 13150), *Pseudomonas aeruginosa* (ATCC 27853) and *Escherichia coli* (ATCC 25922). Several colonies of each microorganism were used to obtain bacterial suspensions in Phosphate-Buffered Saline (PBS) up to a 0.5 McFarland turbidity standard. From these, the following dilutions were made: 1:10, 1:100 and 1:1000. A quote of 50 µL of suspension for each bacteria from the latter dilution was sown and spread on the stethoscope membranes using a swab soaked with sterile PBS, and then dried under an extractor fan. Control membranes were directly cultured in Petri dishes, and the treated ones after a running cycle for each of the two LEDs.

All plates were incubated at 36 °C and read after 48 h by laboratory technicians; results were reported in Colony Forming Units (CFUs)/plate.

### 2.3. Statistical Analysis

Our preliminary assessment (using the Kolmogorov-Smirnov test) was that data did not follow a normal distribution. Therefore all variables were analyzed using the non-parametric Mann–Whitney test to compare the number of CFUs in untreated and treated membranes (i.e., before and after the UVC disinfection) for both phase two and four of the study. Stata^®^ SE, version 12.1 (StataCorp, College Station, TX, USA) was used to perform statistical analysis. A statistical significance level of 99% (*p* < 0.001) was used for all inferential analyses.

## 3. Results

**Figure 2:**
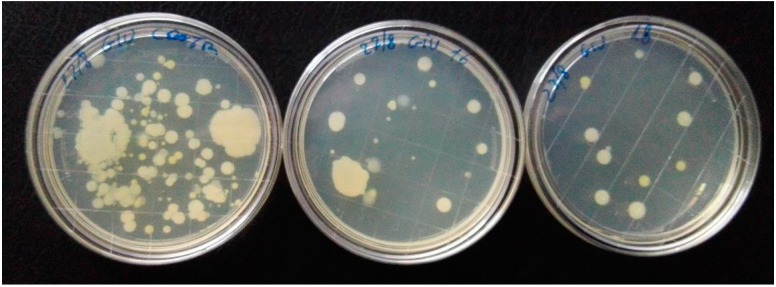
On the left, Petri dish showing baseline volunteer contamination after auscultation. The other two plates show the reduction of the contamination after one cycle (5 min) of UVC disinfection obtained by LED 16 (center plate) and LED 18 (plate on the right). Irradiation was performed after 1668 cycles of LEDs functioning.

After the incubation time, baseline volunteer contamination (control) was 104 CFUs while the result was 15 CFU after irradiation with LED 16, and 12 CFUs after irradiation with LED 18 (Figure 2). Both reductions were statistically significant (*p* < 0.001).

**Figure 3:**
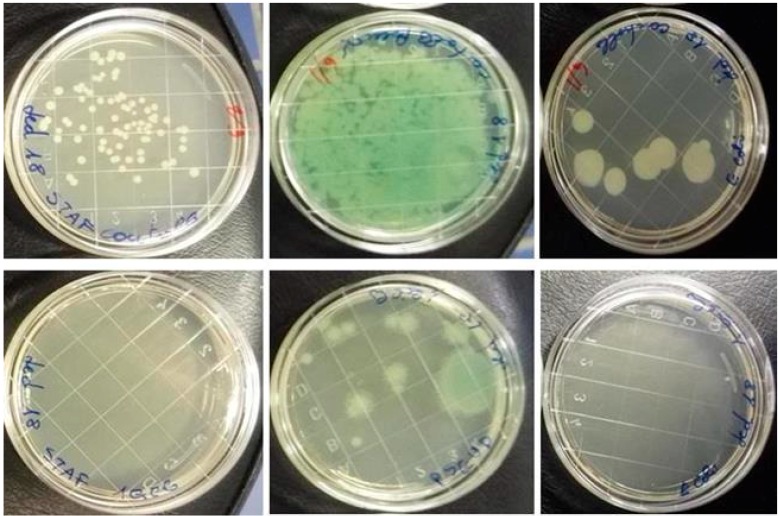
Examples of laboratory contamination before (above) and after (below) UVC treatment: on the left Petri dishes contaminated with *Staphylococcus aureus*, in the middle dishes contaminated with *Pseudomonas aeruginosa*, on the right dishes contaminated with *Escherichia coli*.

Statistically significant differences (*p* < 0.001) were also found in the reduction of laboratory contamination: after treatment with LED 16 and LED 18, no CFUs were observed on treated membranes contaminated with *S. aureus* and *E. coli.* In contrast, on control plates the numbers ranged from 9 to 71 CFUs. *P. aeruginosa* controls showed an uncountable number of CFUs, while treated membranes showed 3 and 12 CFUs for LEDs 16 and 18, respectively (Figure 3).

## 4. Discussion

Our study tested two settings: in the first one we tried to recreate the “real conditions” of use of a stethoscope, simulating a patient who would have been involved in daily clinical activity. We aimed to assess microbial cross-transmission and its reduction after UVC irradiation. Indeed, on the skin, various factors such as perspiration, presence of body hair, sebum and resident bacterial flora could create conditions promoting the contamination of the medical device. Results obtained from this part confirm the presence of bacterial contamination on the stethoscope membrane after the auscultation of a patient. These data are in accordance with literature: a review made by O’Flaherty et al. [3] reported that stethoscopes frequently show microbial contamination and that bacteria can be transferred from the skin to the stethoscope and vice versa. Moreover, Crespo et al. [10] highlighted that the same strain of *Pseudomonas aeruginosa* had been recovered both from patients’ skin and stethoscopes, although it was not detectable on the hands of HCWs employed in the contaminated wards. Similarly, Gastmeier et al. [11] reported that, in an outbreak of *Klebsiella pneumoniae* in a neonatal intensive care unit, the same strain of bacterium, causative agent of bloodstream infections, had been found on stethoscopes belonging to the incubators of two different newborns. We found a reduction in microbial contamination after treatment with UVC LEDs which were previously used for more than 130 h.

In contrast, in the context of the second setting of the study we evaluated the biocidal effect of the UVC LEDs on contaminated stethoscope membranes with specific bacteria often involved in HCAIs [15]. In addition, in this setting it emerged that the UVC light reduces microbial contamination significantly, even after more than 243 h of use, corresponding to the more than 1200 clinical visits in which the stethoscope would be used.

These results are a step forward in assessing the usefulness of a device that implements UVC LED technology for disinfecting the membranes of the stethoscopes. The bacterial reduction we assessed using UVC LED technology fell into the percentage range of the most common method for cleaning stethoscope membranes, i.e., treatment with alcohol (96.3%–99%) [16,17]. UVC light is a physical approach that exerts a germicidal effect by breaking the molecular bond in DNA [18]. It represents a possible solution to the emerging problem of microbial resistance to disinfectants commonly used in healthcare settings [19,20,21] and it is already used for disinfecting patient rooms [22,23,24].

Several physical parameters such as exposition time, distance between light source and irradiation target should be considered and carefully managed when UVC light technology is used to obtain disinfection: for example, the irradiance decreases as the square of the distance. Therefore, minimal changes in one or more of these parameters could lead to substantial variations in disinfection effectiveness.

UVC light is traditionally generated by a mercury bulb lamp while UVC LED technology represents a new emerging approach, for which there is still lack of practical studies and applications. There are many benefits of using UVC LED technology instead of bulb lamps: (i) it is “ecological” because it does not produce hazardous waste products that need to be disposed, such as mercury or xenon; (ii) although UVC LEDs have a shorter lifetime than illumination LEDs, they have longer operative use when compared with mercury lamps; (iii) LEDs have no warm-up time; (iv) there is no degradation of the LEDs performing continuous on/off cycles; (v) the flow architecture is adjustable and the emission wavelength is customizable; (vi) LEDs show great robustness as they are made with a shock resistant semiconductor; (vii) LED technology does not generate heat even after prolonged use; (viii) UVC LEDs may be fully operative even at low voltage.

Because of these characteristics it could be possible to create portable and innovative devices for applications such as stethoscope disinfection. A device which can be worn directly on the coats of HCWs could have multiple advantages. Specifically, because of its wearability the operator would always have access to the device, overcoming some of the most common reasons for poor adherence to good practices of stethoscope disinfection. It could be a reminder of good hygiene practices for the HCWs as well as an indicator for the patients. Another advantage could be its automatic and rapid action: the device could start when a stethoscope head is inserted and it could automatically switch off when removed, also ensuring safety for eyes and skin.

Regarding any possible cost comparisons between the use of an UVC LED device or alcohol in disinfecting stethoscope membranes, there are some considerations to keep in mind: the cost of an UVC LED device could obviously vary due to several aspects (e.g., materials, power, LED/electronics parameters, etc.). However, after the initial investment of buying the device, which has long durability, there is no need for consumables such as alcohol and cotton balls, avoiding further expenditure. In addition, because the device could be created as a wearable tool, it really could contribute to an increased use of the hygienization procedure, addressing a common cause of lack of stethoscope disinfection, i.e., the need for consumable cleaning agents that are not immediately available.

Good hygiene practices could also play a role in the reduction of HCAIs, preventing the spread of bacteria due to this widely used medical device. The implementation could lead to a significant positive impact, both in health and economic terms: HCAIs have an important impact on healthcare systems, generating direct and indirect costs. Direct costs have a specific relationship with the infections themselves (e.g., expenditure for diagnostic exams, treatments and prolongation of hospital stay) [25], while indirect costs are related to productivity and non-medical expenses [26].

UVC LED technology has some limitations, in part due to the inability to produce high output, (a problem that future developments of this technology should minimize), and in part because of the properties of UVC light that are effective only on the surfaces which are irradiated. It also does not remove dust and dirt. Useful precautions to improve disinfection could be to dry the stethoscope membrane and remove visible residual dirt from it before the irradiation. Another problem is how to uniformly disinfect the rim of the stethoscope membrane, although possible solutions can be found and contamination is usually lower than on the full membrane.

The cycle duration of disinfection was set at 5 min: this is a limited time, however, in some medical areas such as an emergency department, while 5 min might be excessive because of the great number of patients to be visited. To overcome this problem several solutions can be implemented, such as providing a more powerful UVC LED or using more than one. Another potential critical aspect of irradiating the stethoscope membrane with UVC is that prolonged use may change the color and the nature of the polymers, although this is also a common problem with disinfectants (e.g., isopropyl alcohol based disinfectants) [27]. The problem is negligible because UVC rays emitted from LEDs are at such low power that it would be very unlikely that they would damage the membrane. Moreover, the periodic replacement of a stethoscope membrane is simple and very cheap and there are even UV-resistant materials on the market. In our experiment we did not find any perceivable changes in the membrane.

This study could have some potential limitations. First, relative to the “real setting”, the sample size is constituted by only one volunteer: it would be interesting to evaluate this technology also in a clinical setting with a larger sample size. Relative to the “laboratory setting”, we use only certain bacterial species. In literature it is reported that other species of bacteria, such as Vancomicin-resitant *Enterococci* (VRE), *Clostridium difficile* and *Acinetobacter* spp. could contaminate the membranes of stethoscopes [3,7,28,29]. Therefore, further studies would be useful for assessing the efficacy of the UVC-LEDs on these species of bacteria also.

## 5. Conclusions

UVC-LEDs demonstrated the capacity to significantly reduce microbial growth on stethoscope membrane after clinical auscultation. This capacity is also confirmed after laboratory contamination of specific microbes that are often involved in HCAIs. These results were obtained after a prolonged time of use of the LEDs involved in the study, demonstrating the effectiveness of this technology in reducing microbial contamination after several working hours. Thanks to the characteristics of this technology, its development could represent a new approach for the improvement of disinfection practices of stethoscopes.
